# Platform Architecture for Decentralized Positioning Systems

**DOI:** 10.3390/s17050957

**Published:** 2017-04-26

**Authors:** Zakaria Kasmi, Abdelmoumen Norrdine, Jörg Blankenbach

**Affiliations:** Institute for Computing in Civil Engineering & Geo Information Systems, Rheinisch-Westfälische Technische Hochschule Aachen University, Mies-van-der-Rohe-Str. 1, Aachen 52074, Germany; norrdine@gia.rwth-aachen.de (A.N.); blankenbach@gia.rwth-aachen.de (J.B.)

**Keywords:** magnetic field positioning system, ultra-wideband positioning system, RIOT-OS, embedded system, microcontroller, STM32F407, LPC2387, TDMA, RTC, DS3234

## Abstract

A platform architecture for positioning systems is essential for the realization of a flexible localization system, which interacts with other systems and supports various positioning technologies and algorithms. The decentralized processing of a position enables pushing the application-level knowledge into a mobile station and avoids the communication with a central unit such as a server or a base station. In addition, the calculation of the position on low-cost and resource-constrained devices presents a challenge due to the limited computing, storage capacity, as well as power supply. Therefore, we propose a platform architecture that enables the design of a system with the reusability of the components, extensibility (e.g., with other positioning technologies) and interoperability. Furthermore, the position is computed on a low-cost device such as a microcontroller, which simultaneously performs additional tasks such as data collecting or preprocessing based on an operating system. The platform architecture is designed, implemented and evaluated on the basis of two positioning systems: a field strength system and a time of arrival-based positioning system.

## 1. Introduction

Nowadays, the accurate localization of a user or an object is indispensable for Location-Based Services (LBS), such as asset tracking, inventory management or routing and navigation. Location sensing systems have become popular and emerged as a vital research area in recent years; therefore, research and commercial products have been developed in academic and industrial contexts. A wide range of technologies is used for positioning; they can be classified into outdoor, indoor, infrastructure-less and infrastructure-based systems. The efficiencies of indoor and outdoor positioning systems differ greatly from each other, due to the fact that indoor surrounding areas raise a challenge for position finding, especially for systems based on wireless technologies, because of factors, such as: signal scattering and attenuation due to the high density of obstacles, multipath reflections from walls and furniture or Non-Line-of-Sight (NLoS) [1]. Infrastructure-less positioning systems do not require any infrastructure (e.g., special hardware in a building). Examples of infrastructure-less methods are video-based (e.g., smart-phone camera [2], robot navigation [3,4]), speech source, inertial sensor or wireless sensor network-based methods. In contrast, infrastructure-based positioning systems need infrastructure such as permanently-installed hardware, electrical power, walls or tripods for mounting the reference points or Internet access [1]. Examples of infrastructure-based positioning systems are ultra-wideband- (UWB) or magnetic field-based localization systems [5,6]. Another example of infrastructure-based positioning solutions is RFID-based indoor localization systems, which can include RFID readers and battery-free tags [7,8]. Generally, the RFID systems enable the localization by using the proximity-based, distance-based [9,10] or the scene analysis [7] methods.

The last two classes of positioning systems can complement each other. Since, on the one hand, the infrastructure-based positioning system can be used as a complementary system in an infrastructure-less positioning system in order to provide a starting point or to support a long-term stability, on the other hand, the infrastructure-less based method can cover areas that cannot be reached from an infrastructure-based method [11].

Another class of positioning systems is the collaborative and device-free localization systems. The collaborative position localization technique is based on various units (sensor nodes) that collaborate to achieve their positions. This technique is also named cooperative or network position location [12]. The collaboration between the sensor nodes has been normally assumed to occur in Wireless Sensor Networks (WSNs) [13]. Device-free positioning systems, which can be used for perimeter security, enable the tracking of users without wearing any devices. Device-free localization systems can use technologies such as pressure or electric field sensors installed under the floor [14].

Therefore, a platform is required for the realization of the previously-mentioned localization systems. The architecture of a platform includes data processing and the system component interaction and design. The data processing can be computed on a server or in a Mobile Station (MS) in the case of the centralized, or decentralized and distributed approach, respectively. The system component can be an MS or an anchor.

To the best of our knowledge, there is neither a standard for the design, nor a detailed description of the architecture of a positioning system. The architecture of most localization systems is roughly described and divided into two parts: the sensor hardware and the positioning algorithm [15]. The first part relies on a variety of technologies, such as electromagnetic waves (e.g., UWB or Wireless LAN (WLAN)), or ultrasound. The positioning algorithm is based on various signal measurement methods, such as Time of Arrival (ToA), signal strength or angle [15].

In this paper, we focus on anchor-based indoor positioning systems. The main contributions of this paper are:

1. The proposition of a layer- and modular-based architecture, which enables the calculation of the localization on-the-fly on the MS. The position is computed on low-cost and resource-constrained devices such as microcontrollers. The proposed platform is non-proprietary and open, since it can be easily extended with other sensors, in order to enable the implementation of positioning systems based on other technologies. The proposed platform can interoperate with existing systems and protocols such as 6LoWPAN.
2. We propose a preprocessing method to remove outliers in measured data, which is convenient for resource-constrained devices, especially in terms of limited stack memory.
3. We introduce a method that calculates the 3D position based on the Singular Value Decomposition (SVD).
4. We propose a pre-processed method for the localization calculation, avoiding the execution of memory and computationally-expensive algorithms such as the SVD or Moore–Penrose, on resource-constrained devices.
5. We also demonstrate the feasibility to deploy the Moore–Penrose algorithm, which is based on SVD, on resource-constrained devices.
6. In order to improve the position estimate, we use the Gauss–Newton, as well as the Levenberg– Marquardt algorithms, which are derived in convenient form for resource-constrained devices.

The proposed localization platform architecture is designed, implemented and tested for two localization systems, a field strength system and a time of arrival-based system. Finally, we evaluate the energy consumption of both systems.

The remainder of the paper is organized as follows: Firstly, we give an architectural overview for localization systems in Section 2. Then, we review related works in Section 3. We introduce in Section 4 a general layered platform architecture for localization systems. In Section 5, we present two examples of the platform architecture. We give an experimental evaluation of both systems in Section 6. Finally, we conclude our paper in Section 7.

## 2. Architectural Overview of Localization Systems

**Figure 1:**
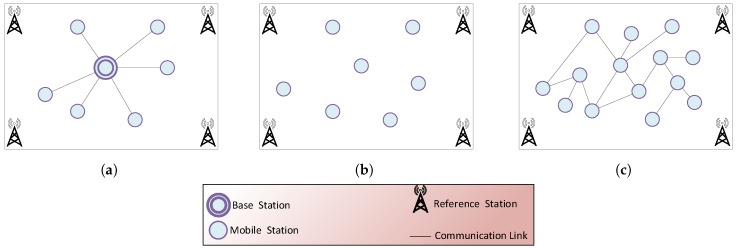
Position evaluation: (**a**) central, (**b**) decentral and (**c**) distributed.

Positioning systems can be classified into three categories: centralized, decentralized and distributed architecture. Figure 1 illustrates different architectures.

The centralized architecture is the most commonly-used one, in which the MSs exclusively communicate with the base station; whereas, the MS modules deliver data collected from sensors to the base station, such as distance or signal measurement. In the decentralized and distributed architecture, both the data processing and the localization calculation take place in the MS, and no data are sent to the base station. Optionally, the result of a position finding could be sent to the base station if necessary. Figure 1b illustrates the case where the position is computed on the MSs and no communication link is used. Furthermore, in a distributed MS network (cooperative evaluation) [16], the MSs can exchange data, to collectively achieve and enhance the accuracy and the efficiency of a localization process [17]. In this case, distributed localization algorithms [18] can be used, which favor cooperative sensing and positioning by involving multiple MSs. The decentralized and the distributed offer the following advantages: the scalability, robustness and energy-awareness.

## 3. Related Work

Firstly, we compare various Indoor Localization Systems (ILSs) from industry, as well as from the research area in terms of the used platforms and architecture. Finally, we give a brief overview of various standards for localization systems.

### 3.1. Centralized Indoor Localization Systems

By the centralized architecture, the MSs uniquely communicate with the base station, which usually has more processing and storage capacity, as well as energy resources than the MSs.

Ubisense has developed a Real-Time Locating System (RTLS) based on the UWB radio technology, which allows a simultaneous tracking of many tags by combining the Angle of Arrival (AoA) and the Time Difference of Arrival (TDoA) methods [19]. The position of the tags is calculated in a location server using the signals transmitted from the tags to the reference points, which are synchronized by timing cable.The Ekahau RTLS is a fingerprinting location system based on the Received Signal Strength Indicator (RSSI), which uses an existing WLAN network in order to enable the tracking of phones, bar code scanners or people wearing WLAN tags [20]. De Angelis et al. proposed a centralized indoor positioning system that is based on alternating current (AC) magnetic fields. The system performs in two phases: the calibration and the trilateration phase [21,22].

The advantages of a centralized architecture include the usage of lightweight MSs, since the whole complexity is shifted to the base station. This enables a high position resolution. The disadvantages comprise single point of failure (e.g., the base station failure), as well as poor performance with a large number of MSs. Therefore, the system can get into energy starvation in the case of continuous communication between the MSs and the base station. Furthermore, the power-saving techniques are hard to implement.

### 3.2. Decentralized Indoor Localization Systems

Most decentralized localization systems are based on smartphones, which use the on-board sensors to calculate the position of a user. Smartphones are significant information interfaces between the user and the environment, which process substantial computational capacity and communication capability [23]. The smartphones incorporate relatively low cost sensors, which do not facilitate an accurate localization. Furthermore, potential energy consumers are the screen, continuous use of the on-board sensors and the communication interfaces. The smartphone-based localization systems can be classified into signal- and inertial-based mobile ILS [24].

The signal-based smartphone ILSs enable the location of users by using existing infrastructure such as a WLAN [25,26]. Most of them are based on fingerprinting, which needs a calibration phase by manually collecting a huge set of training data [27]. In addition, a retraining process is indispensable if the deployment environment is altered. They do not enable continuous (smooth) localization, as well as show limited accuracy due the instability and unreliability of the RSS and the absence of a causal relationship between the Euclidean distance and the RSSI [28].

The smartphone inertial-based ILSs enable the localization of a user by using the on-board magnetometer and accelerometer [29,30]. Inertial-based ILSs allow a continuous localization only for a certain time due to the drift error of the inertial sensors. In addition, compass fluctuations due to a variable magnetic field induced by the indoor environment (e.g., ferromagnetic building materials) lead to inaccurate heading estimation. A start point is essential for the tracking, which requires the use of an external positioning system or the intervention of the user [24]. The accuracy of the localization depends on the smartphone orientation and position with respect to the user’s body [24].

### 3.3. Distributed and Cooperative Localization Systems

Schmid et al. present a proof-of-concept of an ad hoc localization system for persons in a Wireless Sensor Network (WSN) [31]. Yamagushi et al. present two approaches for collaborative indoor localization, which are the stop-and-go localization and the People-Centric Navigation (PCN) methods [13].

Although cooperative ILSs enable the improvement of the location coverage, as well as the location accuracy, especially in the case of poor geometric conditions [12], these systems have to overcome operational requirements, as well as technical challenges [13]; whereby, the operational requirements can be the protection of privacy: users do not allow sharing their positions, or the incentive of users to share their position for, e.g., getting service from the network [32]. Technical challenges include the selection of the reference nodes, the energy efficiency, which requests the optimization of space and time of localization, and the self-localization, particularly by mobile nodes [13]. Another technical challenge is the self-organization due the large numbers of MSs with random environmental characteristics. The error propagation is a serious problem in the distributed evaluation. In addition, most of the multihop localization techniques by the cooperative localization in WSNs are not implemented and are only treated at the theory level; or they were tested in simulated environments [33].

### 3.4. Standards for Localization

**Figure 2:**
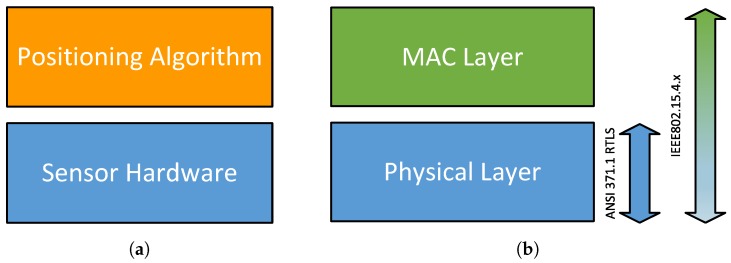
Localization system architectures. (**a**) Industrial and academic area, (**b**) ANSI371.1 RTLS and IEEE 802.15.4.x.

Although there are several positioning systems from the commercial or research fields, the ANSI 371.1 RTLS and the IEEE 802.15.4.x localization standards give only the specification of the physical and media access control (MAC) layer. Figure 2 illustrates this relationship.

The ANSI 371.1 RTLS standard specifies the physical layer for an RTLS, which is called WhereNet and developed by Zebra Technology Company [34]. The WhereNet supports both indoor and outdoor real-time positioning by using the ToA method and the 2.4-GHz Direct Sequence Spread Spectrum (DSSS) PHY [35].

The IEEE 802.15.4a is the first international standard specifying the wireless physical layer, in order to enable precision ranging [36]. The physical layer is based on UWB technology and supports Impulse Radio (IR) UWB and Chirp Spread Spectrum (CSS) [37]. The MAC layer is specified in the IEEE 802.15.4-2003 [38].

## 4. General Architecture of a Platform for Positioning Systems

We use the decentralized architecture due to the on-the-fly capability, robustness, scalability and energy-awareness of the MS.

### 4.1. System Interaction and Components

**Figure 3:**
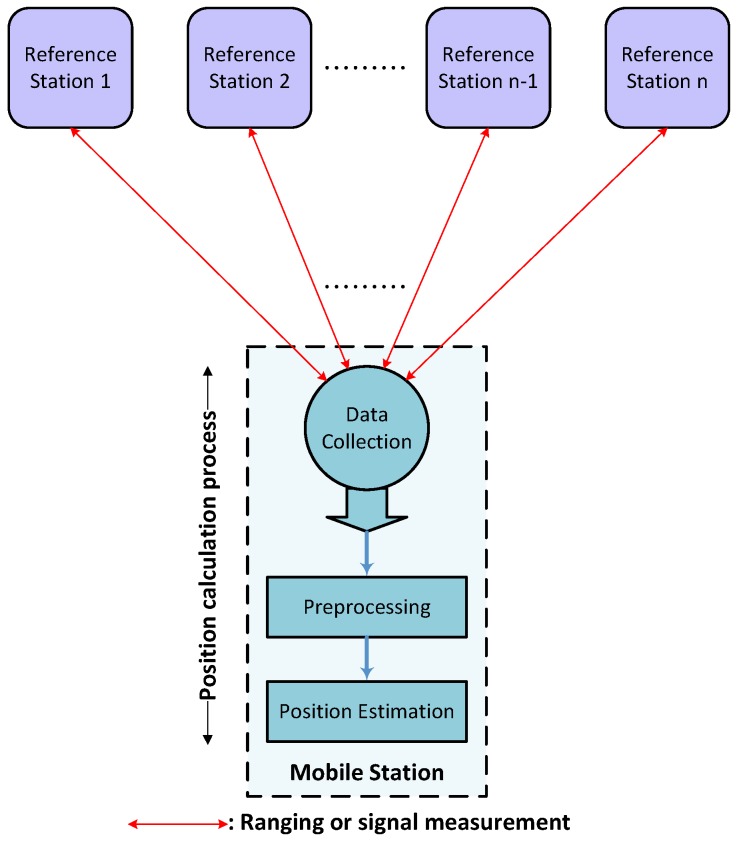
Components’ interaction.

The proposed anchor-based ILS is composed of reference stations (anchors) and an MS, which includes sensors enabling a distance or signal strength measurement. As illustrated in Figure 3, the MS performs a ranging or signal strength measurement of the reference stations, whereby the data, as well as the position computation processing occur on the MS. The localization process consists of three distinct phases: measurement, preprocessing and position estimation. In the first phase, the MS collects data after performing a measurement on the anchors. The measurement data are preprocessed at the second phase, for example to eliminate outliers. Finally, the position is calculated at the third phase.

**Figure 4:**
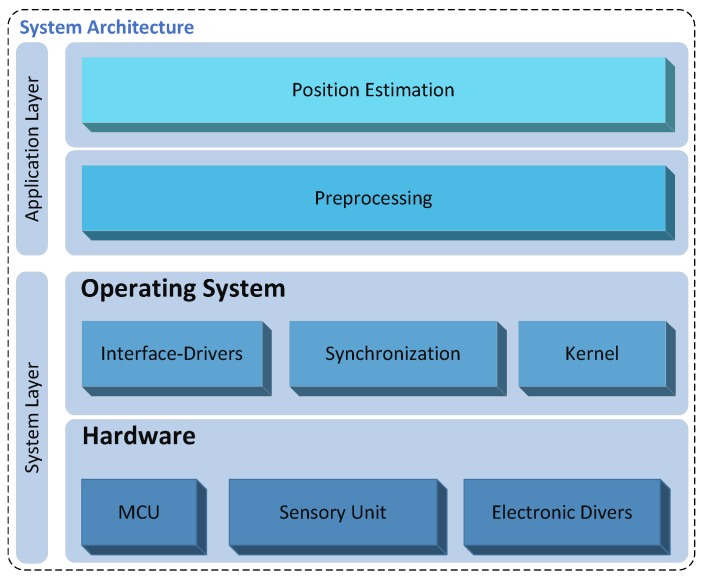
The architecture of the mobile station.

The suggested platform follows a modular-based architecture that ensures the portability and the extensibility of the system. As illustrated in Figure 4, the system architecture of the MS is divided into two layers: the System Layer (SL) and the Application Layer (AL).

### 4.2. System Layer of the Mobile Station

The SL consists of two sublayers: the hardware and the Operating System (OS). Both sublayers will be discussed in the next Section 4.2.1 and Section 4.2.2, respectively.

#### 4.2.1. Hardware Layer

The hardware sublayer includes a computing unit (e.g., Microcontroller Unit (MCU)), sensory unit and electronic drivers. The sensory unit involves sensors such as UWB or magnetometer sensors to accomplish distance or magnetic field strength measurement, respectively. The electronic driver circuits enable, for example, interfacing with external driver units.

#### 4.2.2. Operating System Layer

The OS sublayer includes a software interface driver module, which provides interfaces to communicate with sensors or other devices. Most digital sensors support standardized interfaces such as Serial Peripheral Interface (SPI), Inter Integrated Circuit (I2C) buses and Universal Asynchronous Receiver Transceiver (UART), which encourage the interoperability and the extensibility of the system with other technologies. A synchronization unit can serve to synchronize the received sensor data between a transmitter and receiver.

OSs for resource-constrained devices will be presented in the next Section 4.2.3.

#### 4.2.3. Operating System for Microcontroller

A multithreaded Operating System (OS) is needed, since various tasks are running simultaneously inside an MS. These tasks may run quasi-parallel [39], performing for example a measurement and a processing of unknown position. The OS represents the upper sub-layer of the system layer, which abstracts the hardware sub-layer (cf. Figure 4). An OS can be essentially characterized by the following key design issues:

i kernel structure, which can follow a monolithic model, layered approach or microkernel paradigm,
ii the scheduler and
iii the programming model [40].

Resource-constrained devices such as microcontrollers are characterized by limited computation and storage capacity, as well as battery power. The OSs for resource-constrained devices vary in the architecture, real-time support, scheduling, as well as the programming model and language [41]. Examples for these operating systems are: FreeRTOS [42], TinyOS [43], Contiki [44] and RIOT-OS [40]. Table 1 shows a comparison of various open-source OSs for resource-constrained devices. Although the FreeRTOS supports the most microcontrollers, FreeRTOS does not enable low power management features, like most real-time OSs, since the energy-savings of modern MCUs are platform specific [45,46]. Contiki and TinyOS lack support for real time, as well as some developer-friendly features, such as standard multi-threading and standard C and C++ programming language.

### 4.3. Application Layer

In order to ensure the portability and the extensibility of the system with various applications and positioning algorithms, the AL as the highest level of the proposed platform follows a modular-based architecture. The interoperability of the AL, as well as of the MS can be extended by using an open-standard format, such as the JavaScript Object Notation (JSON) [47], in order to enable data exchange with other devices: for example, data exchange between the MS and applications located on a PC. The AL is subdivided into two sublayers: the preprocessing and position computing sublayers (cf. Figure 4). Both sublayers of the AL will be discussed in the next Section 4.3.1 and Section 4.3.2, respectively.

#### 4.3.1. Preprocessing

The preprocessing sublayer comprises data filtering, which, for instance, reduces the effect of statistical outliers from data delivered from the SL. The outliers can be filtered out by using the mean, the median or the Median Absolute Deviation (MAD) filters [48,49,50]. The preprocessing sublayer can also provide a calibration routine to correct uncertainties in the measured data.

#### 4.3.2. Position Estimation

The top level sublayer provides the algorithmic core that computes the position. Commonly, a positioning algorithm is applied to estimate the unknown position of an MS based on measurements to reference points. The positioning algorithms must satisfy some practical requirements in order to be implemented in a practical system: for example, the algorithm should be robust against noisy measurements, otherwise the performance of the algorithm can decrease drastically. Before we describe some possible algorithms, we briefly introduce several measurement methods along with performance metrics of positioning algorithms.

(a) Measurement methods:
  The common measurement methods are based on signal strength, angular or distance observations, such as Received Signal Strength (RSS), Angle of Arrival (AoA) or Time of Arrival (ToA). The ToA is the most popular measurement technique, which can be estimated by using various ranging techniques, such as the one-way or two-way ToA, and Time Difference of Arrival (TDoA) [51]. Additionally, hybrid measurements can be used for the positioning, such as TDoA/AoA [52] or ToA/RSS [53].
(b) Performance metrics:
  The performance of a positioning algorithm can be evaluated by the following metrics: accuracy, precision, complexity, robustness, scalability, resilience to error and noise, coverage and cost [15].
  - Accuracy metrics: The localization accuracy metric shows how well the ground truth and estimated positions match. There are a number of accuracy metrics such as the Root Mean Square Error (RMSE), the Cumulative Distribution Function (CDF), the Probability Density Function (PDF) or the Frobenius metric (FROB) [54].
  - Cost metrics: A practical evaluation criterion is the cost of an algorithm, which is often a trade-off against accuracy. Common cost metrics are: algorithm complexity, convergence time, power consumption, reference to node ratio [55].

#### 4.3.3. Positioning Algorithms

Positioning algorithms can be classified into two groups: deterministic and probabilistic methods [12]. Deterministic methods determine directly the position based on the measurements by applying the lateration or the least squares method. The lateration is a popular location algorithm, which computes the position of an unknown MS by measuring its distance or angle from multiple reference positions. The algorithm is called trilateration if the number of the reference points is three, otherwise, it is called multilateration. On the other hand, probabilistic methods [56,57], which are known as Bayesian methods, enable the position finding by considering the uncertainty of the measurements [12]. The Bayesian methods proceed in two steps: the prediction and correction steps.

## 5. Examples of a Platform for Positioning Systems

Based on the general discussion of an open platform, we now present two exemplary platforms for positioning systems. The first positioning system is ToA based and makes use of the UWB technology, while the second is RSS based and uses Direct Current (DC)-pulsed magnetic signals. We apply the decentralized architecture for the two positioning systems. The UWB- and the magnetic signal-based positioning systems will be described in Section 5.1 and Section 5.2, respectively.

### 5.1. UWB-Based Positioning System

**Figure 5:**
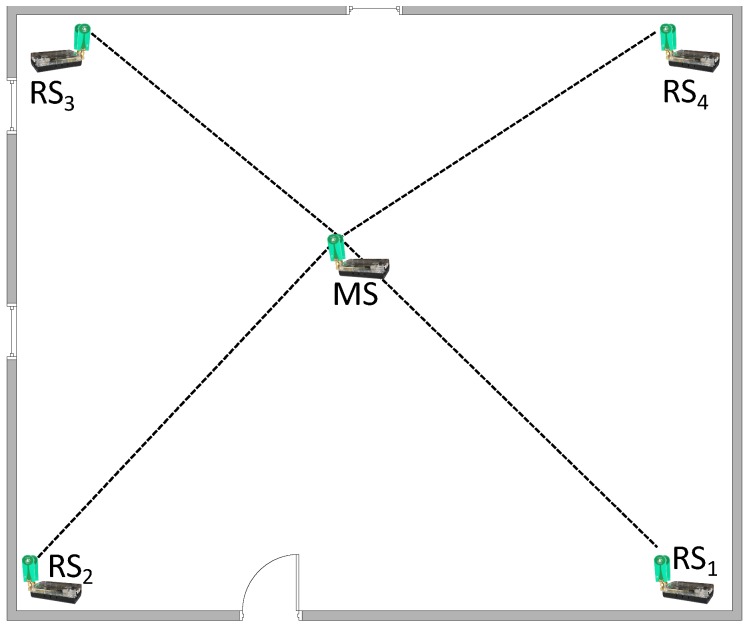
UWB-based Indoor Localization System (ILS) principle. MS, Mobile Station; $RS_{i}$, Reference Station *i*.

The proposed system is composed of various UWB transceivers with known positions as reference stations (RSs) and one MS, which also incorporates a UWB transceiver (see Figure 5). The UWB transceiver computes a peer-to-peer distance to the reference stations using the two-way ToA measurement technique [15]. The position is calculated on the MS based on the measured distances to the RSs.

The system, as well as the application layer of the UWB-based positioning system will be described in the next Section 5.1.1 and Section 5.1.2, respectively.

#### 5.1.1. System Layer of the UWB-Based Mobile Station

**Figure 6:**
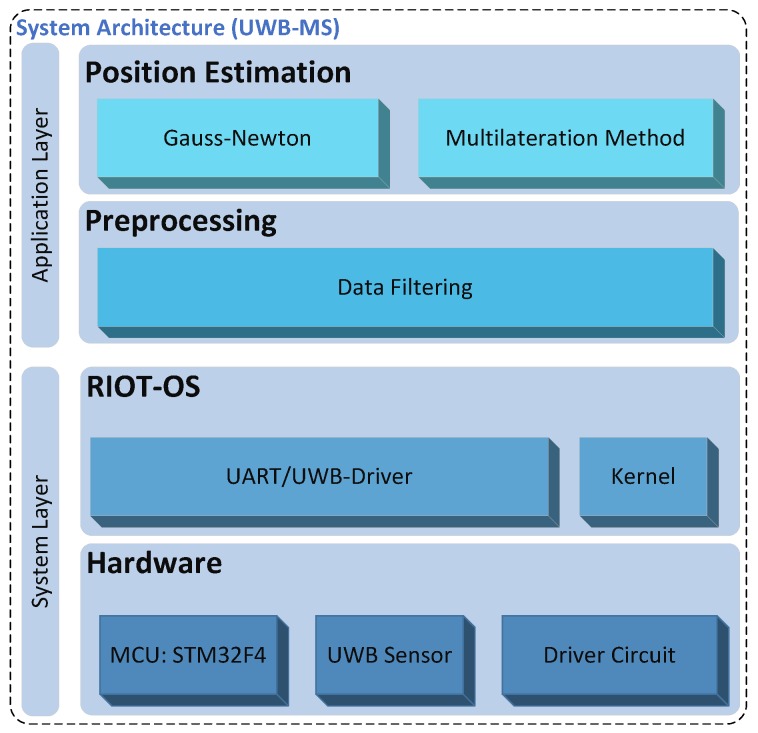
System architecture of a time of arrival-based MS.

As illustrated in Figure 6, the SL is based on the previously discussed template architecture (see Figure 4) and is composed of a hardware and an operating system sublayer, which will be described in this section.

**Figure 7:**
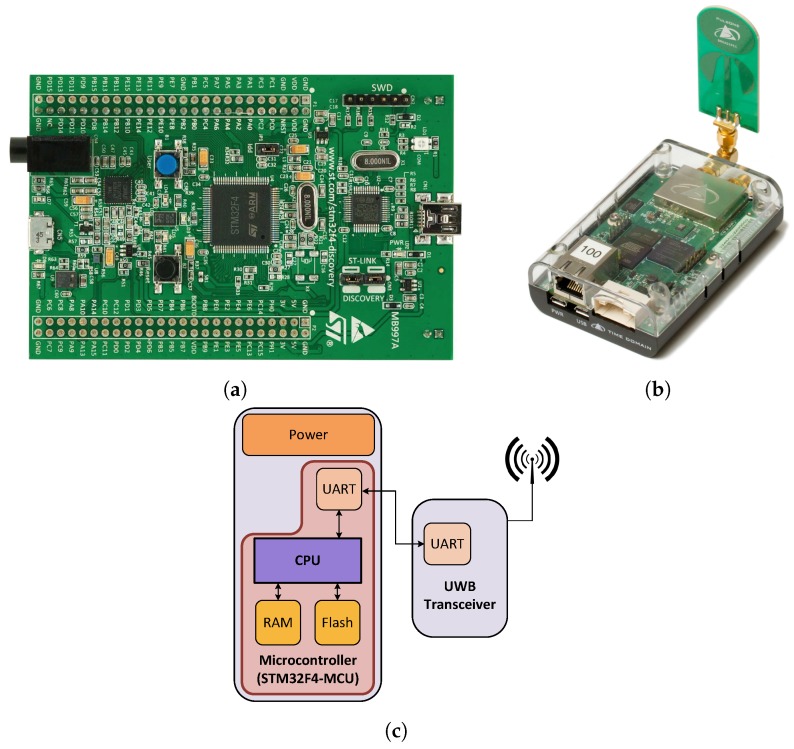
UWB-based localization system hardware. (**a**) STM32F407 Discovery Board [58], (**b**) UWB P440 transceiver [59], (**c**) simplified hardware block diagram.

(a) Hardware:
  The hardware comprises four subsystems: the power unit supplying the sensor board with energy, the Microcontroller Unit (MCU), the sensory unit and the driver circuits (see Figure 7). The hardware layer is implemented based on the STM32F4, ARM Cortex-M4 core operating at 168 MHz and the UWB module P440 ranging sensor from TIME DOMAIN^®^, which enables ranging measurements with an accuracy of a few centimeters. The properties of the used MCU and the UWB ranging sensor are summarized in the Table 2 and Table 3, respectively.
(b) Operating system:
  We use the RIOT-OS [61], which is an open-source IoT operating system developed at the “Freie Universität Berlin”. RIOT-OS is based on a microkernel architecture, which was deployed for a rescue scenario to track and monitor fire fighters. In order to fulfill severe real-time requirements for hard industrial or emergency scenarios, the micro-kernel provides a zero-latency interrupt handling and prioritized threads with a minimum context-switching time. RIOT-OS implements a tickless scheduler, in order to achieve a maximum energy savings and to support deep-sleep mode by all resource-constrained MCUs. Furthermore, the RIOT-OS supports the 6LoWPAN and the IPv6 protocols, which enables the interoperability with existing systems and protocols [62]. Based on the architecture of the RIOT-OS, we developed and integrated device drivers for the P440 ranging sensor and the UART controller. These software driver components build the driver module and are part of the system layer (cf. Figure 6).

#### 5.1.2. Application Layer of the UWB-Based Mobile Station

As mentioned in Section 4.3.1, the preprocessing sublayer serves to remove the outliers in the data delivered from the SL. Therefore, we use the median filter to remove noise from the measured distances captured from the UWB sensor [63]. We apply the shell sort algorithm to implement the median filter, which does not require recursion, such as the quick sort algorithm [64]. Although the iterative shell sort algorithm is slower than the quick sort algorithm, it is suitable for resource-constrained devices such as MCUs with a limited stack size. The AL incorporates a command shell for the interaction with a user or an application by using the serial interface. Furthermore, the data are exchanged by using the JSON between the MS and other systems, such as applications located on a PC. This can enhance the interoperability and is achieved by using a minimalistic JSON parser at the MCU.

In this section, we firstly present an algebraic multilateration approach for the position estimation. Then, we describe two methods to calculate the linear square method for the ranging-based positioning system. Finally, we derive the equations to compute a non-linear least squares (NLS) method in a convenient form for MCUs.

(a) Algebraic multilateration method:
  Assume (x,y,z) and $\left(x_{i},y_{i},z_{i}\right)$ for i=1,2,...,n are the coordinates of the MS and of *n* reference points, respectively. In addition, the measured distances between the reference points and the MS are $d_{i}$. The unknown location of the MS is the intersection of the spheres, whose equations are:
  (1) $${\left(x-x_{i}\right)}^{2}+{\left(y-y_{i}\right)}^{2}+{\left(z-z_{i}\right)}^{2}={d_{i}}^{2}\;\;\;\;\;i=1,...,n.$$
  The system of nonlinear equations in (1) can be solved by different methods [48,65,66,67]. We solved it by transforming the system of equations into a matrix form [68]. The algorithm used is not related to a specific anchor, since most algorithms subtract the coordinates of a specific anchor for the linearization of the equation system. Additionally, the algorithm gives a measure of the solvability of the multilateration problem and provides a recursive least square approach to update the position [68]. The solution of the linearized system is completely determined if the distances from four reference points are known. The problem requires the estimation of the unknown position $\vec{x}=\left(x,y,z\right)$ such that:
  (2) $$A\vec{x}=\vec{b},$$
  where the matrix A and the vector $\vec{b}$ have the following forms [68]:
  (3) $$A=\left(\begin{array}{cccc} 1 & -2x_{1} & -2y_{1} & -2z_{1}\\ 1 & -2x_{2} & -2y_{2} & -2z_{2}\\ 1 & -2x_{3} & -2y_{3} & -2z_{3}\\ \vdots & \vdots & \vdots & \vdots \\ 1 & -2x_{n} & -2y_{n} & -2z_{n} \end{array}\right),$$
  (4) $$\vec{b}=\left(\begin{array}{c} d_{1}^{2}-x_{1}^{2}-y_{1}^{2}-z_{1}^{2}\\ d_{2}^{2}-x_{2}^{2}-y_{2}^{2}-z_{2}^{2}\\ d_{3}^{2}-x_{3}^{2}-y_{3}^{2}-z_{3}^{2}\\ \vdots \\ d_{n}^{2}-x_{n}^{2}-y_{n}^{2}-z_{n}^{2} \end{array}\right).$$
  Equation (2) can be solved based on the QR decomposition in the microcontroller. An efficient method to implement the QR decomposition is the Householder transformation [69]. If A is ill-conditioned or singular, the $\vec{x}$ position can be computed by using the Moore–Penrose pseudo-inverse algorithm [68,69]. The pseudo-inverse is the best linear reconstruction operator in the mean square sense [70], which is more robust and reliable than the Householder transformation, but substantially more computationally expensive:
  (5) $$\vec{x}=A^{+}\vec{b},$$
  whereby $A^{+}$ is the pseudo-inverse of the matrix A [68,69]. The pseudo-inverse matrix can be computed based on the SVD of the matrix A [71]. SVD enables the calculation of the underdetermined and overdetermined systems of linear equations. Furthermore, the SVD is more robust to numerical errors [72], but it is computationally expensive.
(b) Preprocessed pseudo-inverse matrix:
  Since the matrix A in (3) depends only on the coordinates of the RSs, the constant matrix ($A^{+}$) can be computed externally, e.g., in a PC. This method enables saving the resources of the MS, which can be initialized with the matrix $A^{+}$ for, e.g., with the help of a serial communication. In this case, the computation of a new $\vec{x}$ position in Equation (2) is reduced to a matrix multiplication: $A^{+}\vec{b}$.
(c) Non-linear least squares method: Gauss–Newton method:
  The algebraic multilateration method does not always provide a good estimation due to the measurement uncertainties [68]. In this case, the NLS method can be used to improve the position calculated from the algebraic multilateration method. This method is based on the minimization of the squares of the errors:
  (6) $$F\left(x,y,z\right)=\sum_{i=1}^{n}f_{i}^{2}\left(x,y,z\right),$$
  whereby $f_{i}\left(x,y,z\right)$ is the error function:
  (7) $$f_{i}\left(x,y,z\right)=\sqrt{{\left(x-x_{i}\right)}^{2}+{\left(y-y_{i}\right)}^{2}+{\left(z-z_{i}\right)}^{2}}-d_{i}\;\;\;\;\;i=1,...,n.$$
  Minimizing the sum of the square errors is a common problem in the area of applied mathematics, which can be solved, for instance, with the Gauss–Newton or the Levenberg–Marquardt algorithms [73]. We use the Gauss–Newton method to improve the estimated position, which is calculated by using the pseudo-inverse algorithm.
  Since the Gauss–Newton method requires the first derivatives, we define the following Jacobian matrix:
  (8) $$J_{f}=\left(\begin{array}{ccc} \frac{\partial f_{1}}{\partial x} & \frac{\partial f_{1}}{\partial y} & \frac{\partial f_{1}}{\partial z}\\ \frac{\partial f_{2}}{\partial x} & \frac{\partial f_{2}}{\partial y} & \frac{\partial f_{2}}{\partial z}\\ \frac{\partial f_{3}}{\partial x} & \frac{\partial f_{3}}{\partial y} & \frac{\partial f_{3}}{\partial z}\\ \vdots & \vdots & \vdots \\ \frac{\partial f_{n}}{\partial x} & \frac{\partial f_{n}}{\partial y} & \frac{\partial f_{n}}{\partial z} \end{array}\right).$$
  We introduce the error function vector $\vec{f}$:
  (9) $$\vec{f}={\left(\begin{array}{c} f_{1},\;f_{2},\;f_{3},\;\cdots ,\;f_{n} \end{array}\right)}^{T}.$$
  Starting by an initial position guess ${\vec{x}}^{\;(1)}={\left(\tilde{x},\tilde{y},\tilde{z}\right)}^{T}$ calculated from the algebraic multilateration method, the Gauss–Newton method proceeds by the following iterations:
  (10) $$\begin{array}{cc} {\vec{x}}^{\;(k+1)} & ={\vec{x}}^{\;(k)}+{\vec{s}}^{\;(k)} \end{array}$$
  (11) $$\begin{array}{cc} {\vec{s}}^{\;(k)} & =-{\left(J_{f}^{T(k)}J_{f}^{\left(k\right)}\right)}^{-1}J_{f}^{T(k)}{\vec{f}}^{\;(k)}, \end{array}$$
  where ${\vec{x}}^{\;(k)}$ is the *k*-th approximation of the position and ${\vec{s}}^{\;(k)}$ is the *k*-th error correction vector. We calculate $\vec{s}$ by using the Moore–Penrose pseudo-inverse algorithm, since the QR-decomposition, such as the QR-Householder algorithm, can fail due to the singularity or bad conditioning of the matrices.
  Using Equations (7) and (8) leads to:
  (12) $$J_{f}^{T}J_{f}=(\begin{array}{ccc} \sum_{i=1}^{n}\frac{{\left(x-x_{i}\right)}^{2}}{{\left(f_{i}+d_{i}\right)}^{2}} & \sum_{i=1}^{n}\frac{\left(x-x_{i}\right)\left(y-y_{i}\right)}{{\left(f_{i}+d_{i}\right)}^{2}} & \sum_{i=1}^{n}\frac{\left(x-x_{i}\right)\left(z-z_{i}\right)}{{\left(f_{i}+d_{i}\right)}^{2}}\\ \sum_{i=1}^{n}\frac{\left(x-x_{i}\right)\left(y-y_{i}\right)}{{\left(f_{i}+d_{i}\right)}^{2}} & \sum_{i=1}^{n}\frac{{\left(y-y_{i}\right)}^{2}}{{\left(f_{i}+d_{i}\right)}^{2}} & \sum_{i=1}^{n}\frac{\left(y-y_{i}\right)\left(z-z_{i}\right)}{{\left(f_{i}+d_{i}\right)}^{2}}\\ \sum_{i=1}^{n}\frac{\left(x-x_{i}\right)\left(z-z_{i}\right)}{{\left(f_{i}+d_{i}\right)}^{2}} & \sum_{i=1}^{n}\frac{\left(y-y_{i}\right)\left(z-z_{i}\right)}{{\left(f_{i}+d_{i}\right)}^{2}} & \sum_{i=1}^{n}\frac{{\left(z-z_{i}\right)}^{2}}{{\left(f_{i}+d_{i}\right)}^{2}} \end{array}),$$
  and:
  (13) $$J_{f}^{T}\vec{f}={\left(\begin{array}{c} \sum_{i=1}^{n}\frac{\left(x-x_{i}\right)f_{i}}{\left(f_{i}+d_{i}\right)},\;\sum_{i=1}^{n}\frac{\left(y-y_{i}\right)f_{i}}{\left(f_{i}+d_{i}\right)},\;\sum_{i=1}^{n}\frac{\left(z-z_{i}\right)f_{i}}{\left(f_{i}+d_{i}\right)} \end{array}\right)}^{T}.$$
  Equations (12) and (13) are composed of sum terms, which can be implemented in the microcontroller, for example by the use of a for-loop; whereby, the upper bound of the loop is *n*, which is equal to the reference point’s number. The matrix in Equation (12) is symmetrical; this property can be used to reduce the computational burden by computing only the upper or lower part of the matrix. Finally, the terms ${\left(fi+di\right)}^{2}$ and $\frac{f_{i}}{f_{i}+d_{i}}$, which appear in each sum term of the matrix elements, can be computed only once by each iteration in Equations (12) and (13), respectively.

### 5.2. Magnetic Field-Based Positioning System

**Figure 8:**
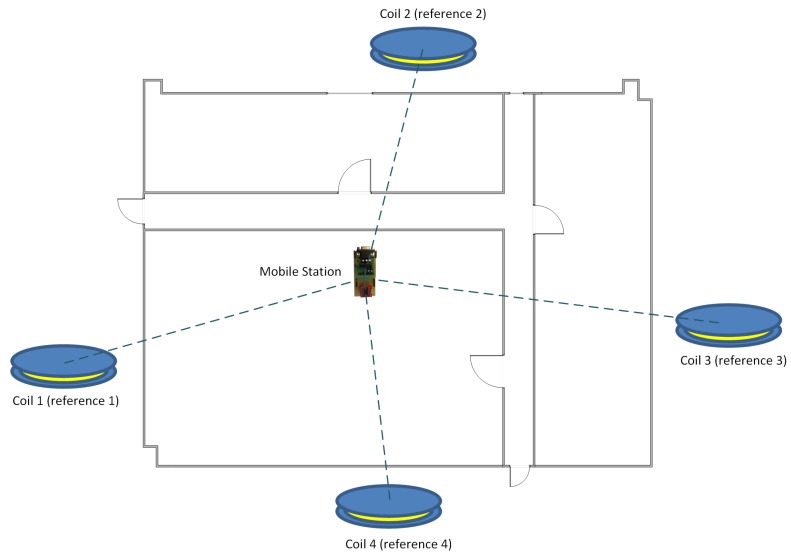
Magnetic indoor local positioning system (MILPS) principle.

The Magnetic Indoor Local Positioning System (MILPS) is a magnetic field based positioning system, which is a representative for an RSS-based localization system [74]. The decentralized MILPS is based on DC-pulsed magnetic signals that show no special multipath effects and have good characteristics for penetrating various obstacle [6]. The MILPS enables a decentralized control of the individual coils (reference stations), as well as the decentralized synchronization of the entire system without the need of communication technology. In our previous work [6], we showed the possibility to synchronize and to control the coils and MS based on low-cost real-time clocks (RTCs). Furthermore, we calculated the distances to the anchors on the MS in a two-dimensional scenario. Based on the previous work, we developed this exemplary platform for MILPS, whereas the MS and the RSs are equipped with real-time clocks, and the MS additionally incorporates a magnetic sensor (see Figure 8). The developed MILPS in this work enables the calculation of an optimized three-dimensional position on the MS based on the measurement of the magnetic field, as well as the elevation angle to the anchors.

**Figure 9:**
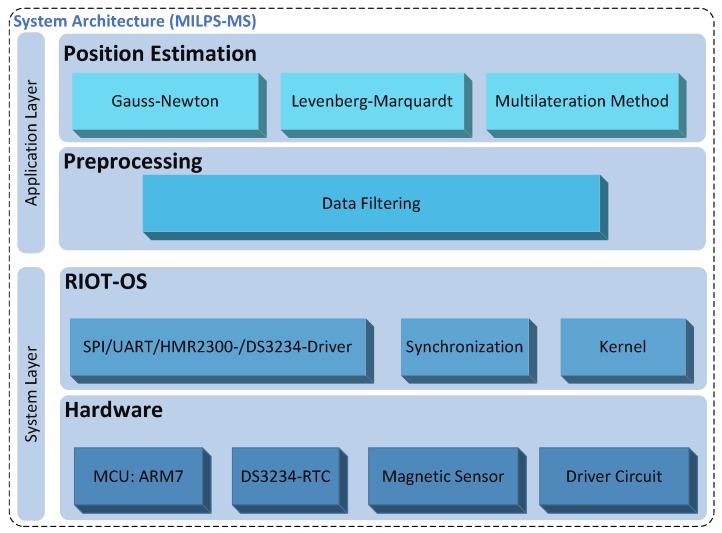
MILPS: System architecture of the Mobile Station (MS).

The system and the application layer of the MILPS, which is illustrated in Figure 9, will be described in the next Section 5.2.1 and Section 5.2.2, respectively.

#### 5.2.1. System Layer of MILPS

Such as the platform template in Figure 4, the MILPS system layer is composed of a hardware and an operating system sublayer, which will be described in this section.

**Figure 10:**
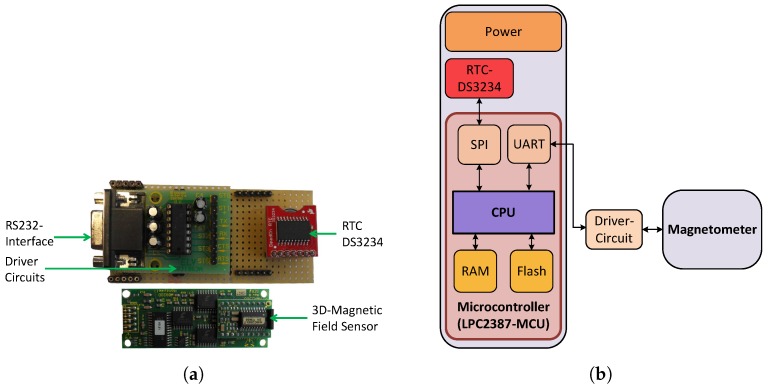
MILPS: Mobile station hardware. (**a**) Mobile station hardware, (**b**) simplified hardware block diagram.

**Figure 11:**
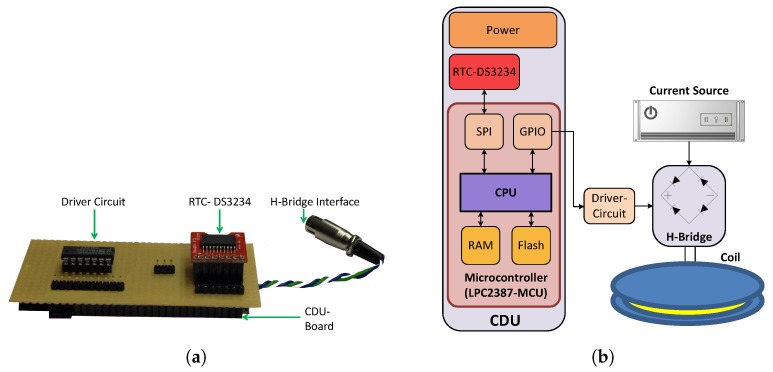
MILPS reference stations hardware. (**a**) Control Driver Unit (CDU), (**b**) simplified hardware block diagram.

(a) Hardware:
  The MILPS has a similar hardware architecture as the UWB-based localization system described before; therefore, the hardware of the MS comprises four subsystems: the power unit, the Microcontroller Unit (MCU), the sensory unit and the driver circuits. The hardware layer is implemented with the LPC2387 ARM7 core (see Table 4). The sensory unit of the MS includes the 3D-magnetic sensor HMR2300, which offers a range of ±2 G [75]. The properties of the used HMR2300 magnetometer are summarized in Table 5. Furthermore, the hardware sublayer of the MS incorporates the DS3234 RTC in order to synchronize the gathered magnetic data, which are generated from the coils. The hardware of the MS is illustrated in Figure 10.
  Each coil is driven via a Control Driver Unit (CDU), which includes a LPC2387-MCU, an RTC and a driver circuit. The driver circuit enables the CDU to interface with an H-bridge, in order to control the voltage polarity. Figure 11 illustrates the CDU, as well as the control of the coils.
(b) Operating system:
  Based on the architecture of the RIOT-OS, we developed and integrated the device drivers for the DS3234 real-time clock and the HMR2300 magnetometer. These software driver components are part of the SL (cf. Figure 9). A decentralized synchronization mechanism enables periodic control of the coils and the MS using the Time Division Multiple Access (TDMA) scheme. At the initialization phase, the RTCs of the MS and the coils are set to the same time. In the operating mode, the coils are activated in fixed duration slots, which are cyclically organized. Simultaneously, the MS gathers the magnetic data from the magnetometer, which can be assigned to the source coils based on the predefined time slots [6].

#### 5.2.2. Application Layer of MILPS

The AL is the highest level of the MS, which follows a modular-based architecture. It is subdivided into two sublayers: the preprocessing and position computing layers (cf. Figure 7b). The first sublayer includes the data filtering module, which uses the the median filter to remove the outliers from the gathered magnetic data, which are delivered from the system layer. The top level sublayer represents the algorithmic core that computes the position on the MS. Both ALs incorporate a command shell for the interaction with a user or an application by using the serial interface, similar to the UWB-based system. Furthermore, the AL of the MS includes a minimal JSON parser. In the following section, we describe the algorithms for the position estimation.

Theoretically, the magnetic field $B_{i}$ generated from the coil *i*, is given by the following equation:

(14) $$B_{i}=\frac{K}{r_{i}^{3}}\sqrt{1+3\sin^{2}\theta_{i}}\;\;\;i=1,2,...,n.$$

In this context, $K=\frac{\mu_{0}NIF}{4\pi }$, where *N* describes the number of turns of the wire, *I* is the current running through the coil, *F* expresses the base area of the coil, $\mu_{0}$ is the permeability of free space, $r_{i}$ is the distance between the MS and coil *i* and $\theta_{i}$ is the MS elevation angle relative to the coil plane [74,77].

In the following subsections, we describe three methods to calculate the position of the MS: the first one is the algebraic multilateration method; the second and third methods are the NLS-based methods, which use the estimated position from the first method as a start value. We used the NLS Gauss–Newton, as well as the Levenberg–Marquardt algorithm, since the algebraic multilateration does not always deliver an optimized position and due to the nonlinear measurement model. Furthermore, we derive the equations to compute these NLS methods in a convenient form for MCUs.

(a) Algebraic multilateration method:
  In the two-dimensional case (2D), when the coil *i* and the magnetometer lay on the same horizontal plane, $\theta_{i}$ is equal to zero. Thus, (14) is reduced to the following equation:
  (15) $$B_{i}=\frac{K}{r_{i}^{3}}\;\;\;i=1,2,...,n.$$
  The position of the MS is computed by using the algebraic multilateration method [68], based on the distances to *n* coils, calculated according to:
  (16) $$r_{i}=\sqrt[{3}]{\frac{K}{B_{i}^{3}}}\;\;\;i=1,2,...,n.$$
  In the general three-dimensional case (3D), the unknown elevation angles $\theta_{i}$ can be estimated by using a three-axis accelerometer, which enables the measurement of the pitch angle β and the roll angle κ of the MS [74]. Based on the measured pitch and roll angles, the elevation angles between the MS and reference stations can be calculated as follows:
  (17) $$\theta_{i}=\arctan\left[-\frac{3}{4}\tan I_{i}\pm \sqrt{{\left(\frac{3}{4}\tan I_{i}\right)}^{2}+\frac{1}{2}}\right],$$
  whereby $I_{i}$ is the inclination of the magnetic field from coil *i*, which is calculated using:
  (18) $$I_{i}=\arcsin\left[\frac{-B_{x^{\prime },i}\sin\beta +B_{y^{\prime },i}\cos\beta \sin\kappa +B_{z^{\prime },i}\cos\beta \cos\kappa }{B_{i}}\right],$$
  where $B_{x^{\prime },i}$, $B_{y^{\prime },i}$ and $B_{z^{\prime },i}$ are the magnetic field components in the coordinate system of the sensor, which is integrated in the MS, and $B_{i}=\sqrt{B_{x^{\prime },i}^{2}+B_{y^{\prime },i}^{2}+B_{z^{\prime },i}^{2}}$ is the magnetic field magnitude. Hence, the distances $r_{i}$ between the MS and the reference stations can be calculated based on the estimated elevation angles $\theta_{i}$:
  (19) $$r_{i}=\sqrt[{3}]{\frac{k\sqrt{1+3\sin^{2}\theta_{i}}}{B_{i}}}\;\;\;i=1,2,...,n.$$
  In this case, the position of the MS can also be calculated by using the algebraic multilateration algorithm. Therefore, similar to the UWB-based localization system, the pseudo-inverse matrix can be processed in a computing unit such as a PC or a laptop, in order to initialize the MCU with the preprocessed result. In this way, the MCU has only to compute a matrix multiplication, in order to estimate an MS’s position.
(b) Gauss–Newton algorithm:
  Based on (16), the (x,y,z) coordinates of the MS can be computed by solving the following nonlinear system of equations:
  (20) $$f_{i}\left(x,y,z\right)=\frac{k}{r_{i}^{3}}\sqrt{1+3\sin^{2}\theta_{i}}-B_{i}=0\;\;\;i=1,2,...,n$$
  whereby, $r_{i}=\sqrt{{\left(x-x_{i}\right)}^{2}+{\left(y-y_{i}\right)}^{2}+{\left(z-z_{i}\right)}^{2}}$, $\sin\theta_{i}=\frac{z-z_{i}}{r_{i}}$; $\left(x_{i},y_{i},z_{i}\right)$ and (x,y,z) are the coordinates of the *i*-th coil and the MS, respectively. $B_{i}$ is the measured magnetic field strength.
  For simplicity, we set u=(x,y,z); the Gauss–Newton algorithm iteratively finds the best estimate $\hat{u}$, which minimizes the sum of squares:
  (21) $$\hat{u}=arg\min_{u}\sum_{i=1}^{n}{\left(f_{i}\left(u\right)\right)}^{2}.$$
  The Gauss–Newton method starts with an initial guess ${\vec{x}}^{\;(1)}$ calculated by the direct method and proceeds iteratively (see Equation (10)) [73,78]; where $J_{f}$ is the Jacobian matrix of the function $f=(f_{1},f_{2},...,f_{n})$ at $u_{k}$. The Jacobian matrix $J_{f}$ is calculated based on Equation (20) to:
  (22) $$J_{f}=\left(\begin{array}{ccc} \frac{\partial f_{1}}{\partial x} & \frac{\partial f_{1}}{\partial y} & \frac{\partial f_{1}}{\partial z}\\ \frac{\partial f_{2}}{\partial x} & \frac{\partial f_{2}}{\partial y} & \frac{\partial f_{2}}{\partial z}\\ \vdots & \vdots & \vdots \\ \frac{\partial f_{i}}{\partial x} & \frac{\partial f_{i}}{\partial y} & \frac{\partial f_{i}}{\partial z}\\ \vdots & \vdots & \vdots \\ \frac{\partial f_{n}}{\partial x} & \frac{\partial f_{n}}{\partial y} & \frac{\partial f_{n}}{\partial z} \end{array}\right),$$
  whereby, $\frac{\partial f_{i}}{\partial x}$, $\frac{\partial f_{i}}{\partial y}$ and $\frac{\partial f_{i}}{\partial z}$ are respectively equal to:
  (23) $$\frac{\partial f_{i}}{\partial x}=-3K\Delta x_{i}\left[\frac{{\left(\Delta x_{i}\right)}^{2}+{\left(\Delta y_{i}\right)}^{2}+5{\left(\Delta z_{i}\right)}^{2}}{dm_{i}}\right],$$
  (24) $$\frac{\partial f_{i}}{\partial y}=-3K\Delta y_{i}\left[\frac{{\left(\Delta x_{i}\right)}^{2}+{\left(\Delta y_{i}\right)}^{2}+5{\left(\Delta z_{i}\right)}^{2}}{dm_{i}}\right],$$
  and:
  (25) $$\frac{\partial f_{i}}{\partial z}=-12K\left[\frac{{\left(\Delta z_{i}\right)}^{3}}{dm_{i}}\right],$$
  where $\Delta x_{i}=x-x_{i}$, $\Delta y_{i}=y-y_{i}$, $\Delta z_{i}=z-z_{i}$ and $dm_{i}\;=\;{\left({\left(\Delta x_{i}\right)}^{2}+{\left(\Delta y_{i}\right)}^{2}+4{\left(\Delta z_{i}\right)}^{2}\right)}^{\frac{1}{2}}{\left({\left(\Delta x_{i}\right)}^{2}+{\left(\Delta y_{i}\right)}^{2}+{\left(\Delta z_{i}\right)}^{2}\right)}^{3}$.
  The iteration process stops when the updates become sufficiently small. Furthermore, the initial guess ${\vec{x}}^{\;(1)}$ is calculated by using the algebraic multilateration method and the distances $r_{i}$ as described in the first method. Similar to the UWB-based ILS, the Gauss–Newton algorithm uses the Moore–Penrose to calculate the error correction in (10), as well as to choose the ${\vec{x}}^{\;(k)}$ with the minimum error value.
  If the elevation angle $\theta_{i}$ is unknown, it can be approximated by replacing the term $\sqrt{1+3\sin^{2}\theta_{i}}$ by “1.5” in (19) [77] or by using an iterative algorithm [79].

1. Levenberg–Marquardt method:
  The Levenberg–Marquardt method is also an algorithm for solving the NLS problems that is based on the trust-region approach [73]. The advantage of the trust-region strategy is the stability against the rank-deficiency of the Jacobian matrix $J_{f}$, which is one of the weaknesses of the Gauss–Newton method [73]. Like the Gauss–Newton method, the Levenberg–Marquardt method proceeds iteratively (see Equation 10), whereby the error correction vector is equal to:
  (26) $${\vec{s}}^{\;(k)}=-{\left(J_{f}^{T(k)}J_{f}^{\left(k\right)}+{\mu^{2}}^{\left(k\right)}I\right)}^{-1}J_{f}^{T(k)}{\vec{f}}^{\;(k)},$$
  where I is the identity matrix and μ is the damping parameter. The error vector $\vec{s}$ is calculated by using the QR-Householder instead of the Moore–Penrose pseudo-inverse algorithm, since it is less computing time consuming, and the Levenberg–Marquardt method is robust against the rank-deficiency of the Jacobian matrix $J_{f}$.
  The initial damping-parameter $\mu^{\left(1\right)}$ can be calculated based on the matrix $A^{\left(1\right)}$
  (27) $$A^{\left(1\right)}=J_{f}^{T(1)}J_{f}^{\left(1\right)},$$
  as follows:
  (28) $$\mu^{\left(1\right)}=\tau \cdot max_{i}\left\{a_{ii}^{\left(1\right)}\right\},$$
  where $a_{ii}^{\left(1\right)}$ are the diagonal elements of the matrix $A^{\left(1\right)}$ and τ is chosen by the user. As a rule of thumb, a small value of τ should be chosen (e.g., $\tau =10^{-6}$), if the initial guess ${\vec{x}}^{\;(1)}$ is believed to be a good approximation; otherwise, $\tau =10^{-3}$ or τ=1 can be used. Furthermore, the value of $\mu^{\left(k\right)}$ can be updated based on the gain ratio ϱ [80]:
  (29) $$\begin{array}{cc} \varrho & =\frac{G^{\left(k\right)}-G^{\left(k+1\right)}}{G^{\left(k\right)}-{\vec{g}}^{\;T^{\left(k\right)}}{\vec{g}}^{\;(k)}}, \end{array}$$
  (30) $$\begin{array}{cc} G^{\left(k\right)} & ={\vec{f}}^{\;T^{\left(k\right)}}{\vec{f}}^{\;(k)}, \end{array}$$
  (31) $$\begin{array}{cc} G^{\left(k+1\right)} & ={\vec{f}}^{\;T^{\left(k+1\right)}}{\vec{f}}^{{}^{\;(k+1)}}, \end{array}$$
  (32) $$\begin{array}{cc} {\vec{g}}^{\;(k)} & ={\vec{f}}^{\;(k)}+J_{f}^{\left(k\right)}{\vec{s}}^{\;(k)}. \end{array}$$
  (33) $$\mu^{\left(k+1\right)}=\left\{\begin{array}{cc} 2\cdot \mu^{\left(k\right)} & \;\;\;\mathrm{if}\;\varrho \leq \;\beta_{0}\\ \frac{\mu^{\left(k\right)}}{2} & \;\;\;\mathrm{if}\;\varrho \geq \beta_{1} \end{array}\right.,$$
  where by $0<\beta_{0}<\beta_{1}<1$ (e.g., $\beta_{0}=0.2$ , $\beta_{1}=0.8$).

## 6. Experimental Evaluation

In this section, we present the results of the experimental evaluation of the two presented positioning systems in Subsection 6.2 and Subsection 6.3, respectively. The aim of the this evaluation is to demonstrate the feasibility to implement the proposed platform; therefore, we will not address issues, such as the impact of the MS or the anchors’ placement and selection on the localization accuracy. These issues are treated in [12,81,82]. For the evaluation, we give a brief summary of the complexity of the used algorithms, as well as the results of the accuracy measurements of the UWB-based system and MILPS. Furthermore, we evaluated the computing time of the algorithms on the STM32F407 and the LPC2387 MCUs, which are running at 168 MHz and 72 MHz, respectively. Finally, we evaluated the energy consumption of the algorithms on both systems as well as of the UWB-based ILS and the MILPS in Section 6.4.

### 6.1. Complexity of the Algorithms Used

The algorithms used in this paper are based on the matrix multiplication, the Moore–Penrose and the QR-Householder algorithm, and their complicity is summarized in Table 6; whereby, *m* and *n* are the number of rows and columns of the matrix A, respectively, while, *n* and *p* are the number of rows and columns of the matrix B, respectively.

### 6.2. UWB-Based Localization System Evaluation

**Figure 12:**
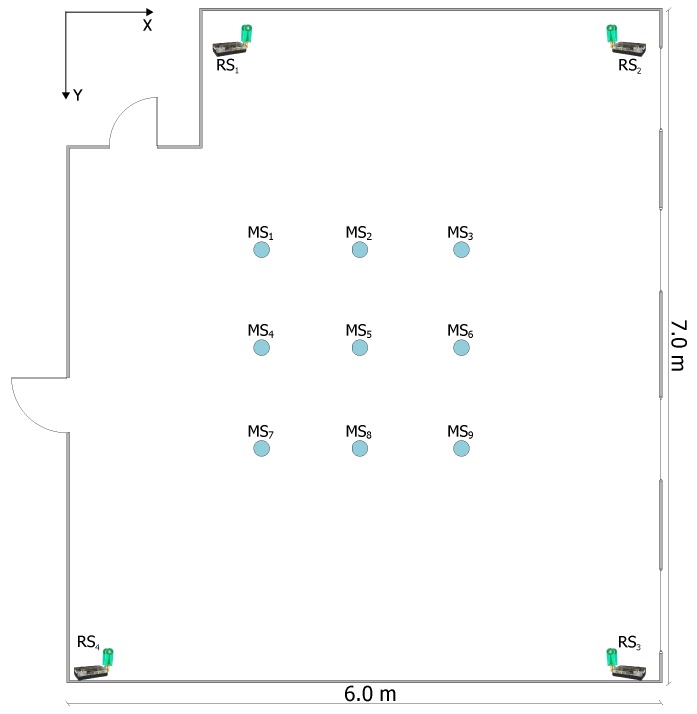
Experimental setup for position measures between various MSs and four reference stations.

A static measurement setup was performed, in order to inspect the positioning performance of the UWB system, whereby four UWB reference transceivers were located in the corners of a 6 m × 7 m room and the UWB mobile stations are placed in several points and at three different heights in the room. Hence, the location of the MS is measured at 27 different locations, whereby the measurement is repeated fifty times at each location. The MSs lie in one meter grid (see Figure 12).

#### 6.2.1. Accuracy Evaluation

**Figure 13:**
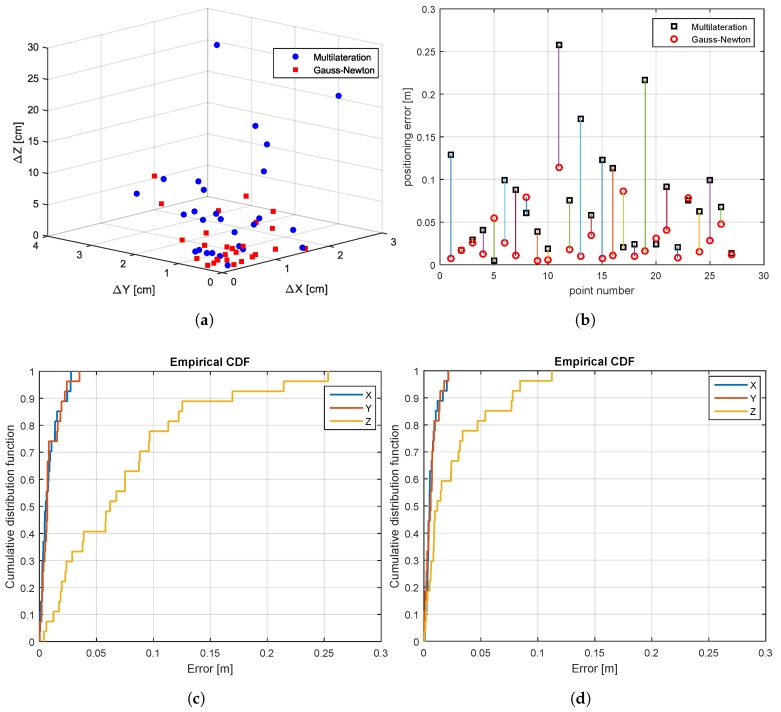
UWB-based system: scatter plots, position errors, and empirical CDFs. ML, Multilateration; GNM, Gauss–Newton Method. (**a**) Scatter plot of the ML and the GNM, (**b**) position error of the ML and the GNM, (**c**) CDF of the points estimated by the ML, (**d**) CDF of the estimated positions after GNM.

Figure 13a illustrates the three-dimensional position errors by using the algebraic multilateration and the Gauss–Newton methods. Figure 13b shows the positioning error, which is defined as the Euclidean distance between the estimated and true position. Figure 13c contains the empirical CDF of the position error of all locations by using the algebraic multilateration method; the error in the x and y coordinates is less than 3.5 cm, while the error in the z coordinate is less than 25.3 cm. The error in the z coordinate results from the unfavorable geometrical configuration, since the reference stations are located approximately at the same height. By further applying the Gauss–Newton method, the positioning error is reduced to 2.2 cm in the x and y coordinate, as well as to 11.2 cm in the z coordinate (see Figure 13d).

#### 6.2.2. Computing Time Measurement

The $A^{+}$ matrix is calculated based on the Moore–Penrose method, and only once, in the initial phase at the start of the MCU or the positioning application. Based on the computed $A^{+}$ matrix, the localization of the MS is determined by the algebraic multilateration method. The position of the MS can be improved by using the Gauss–Newton algorithm, which uses the position delivered from the algebraic multilateration method as a starting point. The Gauss–Newton proceeds iteratively up to the desired accuracy or the maximal iteration number is reached. The average iteration number in this experiment is five. Despite the constrained computing resources of the STM32F407 MCU, the computing time of each positioning is in the order of 0.032 ms without using the Gauss–Newton algorithm. In contrast, the mean estimated position time increases approximately up to 7.9 ms by using the Gauss–Newton algorithm. The evaluation of the described computing steps is summarized in Table 7.

### 6.3. MILPS Evaluation

**Figure 14:**
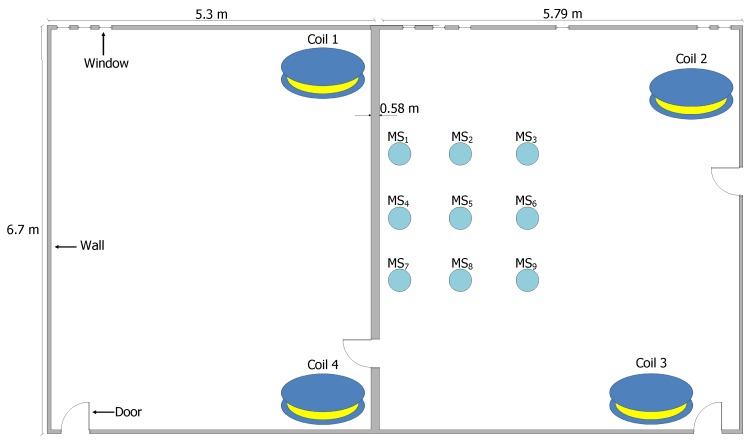
Experimental setup for position measures between various MSs and four coils.

For the computing time and the accuracy evaluation of the MILPS, we placed four coils inside two rectangular rooms. The two rooms are separated by a wall, which is 0.58 m thick (see Figure 14). Two coils are placed in a room of a surface of 5.3 × 6.7 m${}^{2}$, while the other coils are placed in a room of a surface of 5.79 × 6.7 m${}^{2}$. The MSs are placed in 27 various positions, since each MS represented in Figure 14 is located at three different heights of 0.655 m, 1.423 m and 2.308 m. The true positions of the reference and mobile stations are determined by geodetic methods with millimeter accuracy using a tachymeter. We choose this configuration to demonstrate that the MILPS can measure the position even if the coils and the MSs are separated by walls.

#### 6.3.1. Accuracy Measurement

**Figure 15:**
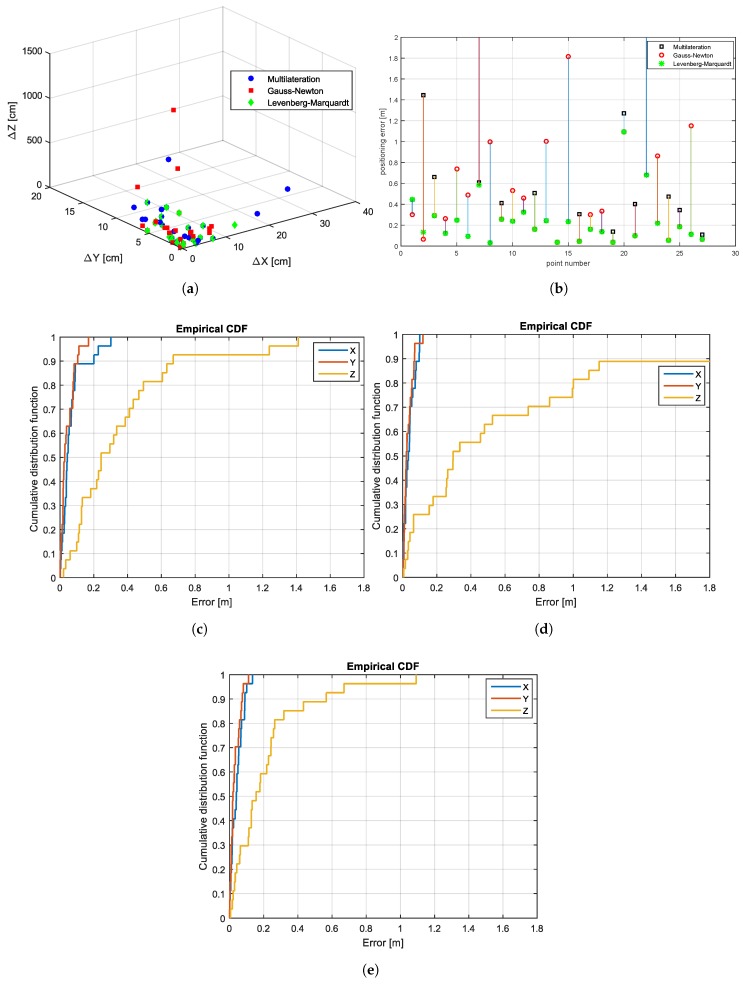
MILPS: scatter plots, position errors, and empirical CDFs. GNM, Gauss–Newton Method; ML, Multilateration; LVM, Levenberg–Marquardt Method. (**a**) Scatter plot of the ML, the GNM and the LVM, (**b**) position error of the ML, the GNM and the LVM, (**c**) CDF of the points estimated by the multilateration, (**d**) CDF of the estimated positions after GNM, (**e**) CDF of the estimated positions after LVM.

Figure 15 presents a comparison between the algebraic multilateration, Gauss–Newton and Levenberg–Marquardt methods in terms of errors, which are illustrated in Figure 15a. Figure 15b shows the positioning error, which is defined as the Euclidean distance between the estimated and true position. As illustrated by this figure, the position errors of Point Numbers 7 and 22 are out of the bound by the Gauss–Newton algorithm, since it diverges. Figure 15c shows the experimental results of the positioning error obtained from the algebraic multilateration method, which are represented by the CDF. In this example, the error in the x and y components is lower than 30 cm. However, the z component of the MS coordinates shows the worst performance, since three coils were placed at nearly equal heights.

The application of the Gauss–Newton method reduced the errors in the x and y coordinates to 10 cm, but impairs the z coordinate values (see Figure 15d). In contrast, by using the Levenberg–Marquardt method, the errors in the z coordinates are limited to 0.6 m for 93% of the measured points and to 1.1 m for other points (see Figure 15e). Furthermore, Figure 15a shows that the Levenberg–Marquardt method has generally lower deviations in all coordinate components compared with other methods.

#### 6.3.2. Computing Time Measurement

As explained in Section 5.2.2a, we used the algebraic multilateration method to estimate the position of the MSs illustrated in Figure 14. Therefore, the $A^{+}$ matrix is also calculated based on the Moore–Penrose method, only once, at the start of the MCU. Similar to the UWB-based system, the position estimation of the MSs can be optimized by using the Gauss–Newton or the Levenberg–Marquardt algorithms. Both algorithms utilize the position carried out by the algebraic multilateration as a starting point and proceed iteratively up to the desired accuracy or when the maximal iteration number is reached. The average iteration number in this experiment is seven and six for the Gauss–Newton and the Levenberg–Marquardt method, respectively. The mean time of calculating a starting position and the computing resource-constrained LPC2387-MCU is approximately 0.1 ms. The computing time of an estimated position increases approximately up to 33 ms or 21 ms by using the Gauss–Newton or the Levenberg–Marquardt algorithm, respectively. The evaluation of the described computing steps and the used NLS methods is summarized in Table 8.

### 6.4. Energy Consumption

The energy consumption of the algorithms is measured based on the measurement of the drain-source current in the supply line, which is powered by a reference voltage supply $V_{cc}$ ($V_{cc}=5$ V). Hence, the energy used for each localization processing task can be calculated by integrating the electric power over the times, which are summarized in Table 7 and Table 8. We measured a current consumption of about 75 mA and 69 mA at the ambient temperature of $26{\;}^{\circ }$C for the STM32F4 and the LPC2387 MCU in the active mode, respectively. The measured energies for the localization algorithms by the UWB-based ILS and the MILPS are summarized in Table 9.

We also measured the energy consumption of the MS performed for the UWB-based ILS, as well as for the MILPS by using the aforementioned method for the energy consumption of the algorithms. We measured a current consumption of about 410 mA and 27 mA by the UWB transceiver and the magnetometer, respectively; whereas, the measurement times by the UWB-ILPS, as well as by the MILPS are 120 ms and 1 s, respectively. The total energies, which are required for a position estimation, are calculated based on the current drain of the sensors, as well as the energy consumption of the MCU (see Table 9). The energy usages of the UWB-based ILS and the MILPS are summarized in Table 10, whereby the energy consumptions of the UWB transceiver and the magnetometer are 246 mWs and 405 mWs, respectively. The UWB solution is more energy-efficient than MILPS, since the UWB sensor measures much faster than the magnetic sensor (approximately 9x).

## 7. Conclusions

In this article, we present a platform for indoor location systems that is designed and implemented for a decentralized architecture, as well as tested for two positioning technologies. The investigated systems use the UWB and magnetic technologies with different measurement methods, which are the time of arrival and the field strength techniques, respectively. The suggested platform is modular and layer based, in order to enable a better reusability of the software components and extensibility with various localization technologies and algorithms. The interoperation with other systems can be reached by using standardized interfaces and data format. Furthermore, the use of an IoT-capable OS enables the MS to be a part of the IoT network. The presented platform can be also a basis for a distributed localization approach.

## Figures and Tables

**Table 1:** Comparison of operating systems. FIFO, First-In First-Out; TOS, TinyOS.

OS	Architecture	Real Time	Scheduling	Programming Model	Programming Language
FreeRTOS	Monolithic	Full Support	Round-robin preemptive and cooperative	Threads	C
TinyOS	Monolithic	No Support	FIFO	Primarily event driven, support for TOS threads	nesC
Contiki	Modular	Partial Support	Event based	Protothreads and events	C with some constraints
*RIOT-OS*	*Microkernel*	*Full Support*	*Tickless, preemptive scheduling with priorities*	*Threads*	*C and C++*

**Table 2:** The properties of the STM32F407 MCU. MCU, Microcontroller Unit [60].

MCU	Family	Vendor	Frequency	RAM	Flash
STM32F407	ARM Cortex-M4	ST Microelectronics	168 MHz	192 KB	1024 KB

**Table 3:** The properties of the deployed P440 ranging sensor [59].

Accuracy	Max. Operating Range	Max. Ranging Rate	Frequency Range	Transmission Power
2.1 cm	300 m–1100 m	125 Hz	3.1 GHz–4.8 GHz	50 μW

**Table 4:** The properties of the deployed LPC2387 MCU. [76].

MCU	Family	Vendor	Frequency	RAM	Flash
LPC2387	ARM7	NXP	72 MHz	96 KB	512 KB

**Table 5:** The properties of the HMR2300 magnetometer. FS, Full Scale [75].

Range	Sample Rate	Resolution	Accuracy
±2 Gauss (G)	up to 154 Hz	up to 70μG	0.5% FS (over ±1 G)

**Table 6:** Complexity of various algorithms.

Algorithm	Complexity (Flops)	
Matrix multiplication: $A_{m,n}\times B_{n,p}$	mp(2n−1)	[83]
QR-Householder	$2mn^{2}-\frac{2}{3}n^{3}$	[84]
Moore–Penrose pseudoinverse	$14mn^{2}+\frac{16}{3}n^{3}$	[85]

**Table 7:** Mean computing times of the algorithms used by the UWB-based System. Computing times measured on an STM32F407 running at 168 MHz.

Algorithm	Computing Time [μs]
$A^{+}$ for the multilateration method (at the start)	2115
Multilateration method	32
Gauss–Newton per iteration	1561

**Table 8:** Mean computing times of the used algorithms by MILPS. Computing times measured on an LPC2387 running at 72 MHz.

Algorithm	Computing Time (μs)
$A^{+}$ for the multilateration method (at the start)	4563
Multilateration method	92
Gauss–Newton method per iteration	4645
Levenberg–Marquardt method per iteration	3467

**Table 9:** List of measured energy consumption values of the algorithms by the UWB-based ILS and MILPS.

Algorithm	UWB-Based ILS Energy (μWs)	MILPS Energy (μWs)
$A^{+}$ by the multilateration method (at the start)	793.13	1574.24
Multilateration method	12	31.74
Gauss–Newton per iteration	585.38	1602.53
Levenberg–Marquardt method per iteration	−	1196.12

**Table 10:** List of measured energy consumption of the UWB-based ILS and the MILPS for a position estimation.

Localization System	Energy (mWs)
UWB-based ILS	246+0.793+0.012+5×0.585≃249.73
MILPS (Gauss–Newton)	405+1.6+0.032+7×1.63≃418.04
MILPS (Levenberg–Marquardt)	405+1.6+0.032+6×1.21≃413.89
